# New Diseases

**Published:** 1916-02

**Authors:** 


### New Diseases.

There is always something new under the sun in the way of diseases. A catalogue of the diseases of this decade would look like a Latin lexicon to one of the old family practitioners of fifty or sixty years ago, could he come to life and inspect it. Even ten or five years makes wonderful changes. For instance, chauffeurs who crank their cars often suffer a fracture of the arm or a peculiar strain in a way seldom seen until the automobile came into use. Telegraphy spasm, a complaint caused by the continued operation of telegraph instruments, and resulting in the hand becoming stiff and uncontrollable, is another trouble with which physicians have to contend now. Air-sickness, resulting from flying in different stratas of air, is something of very recent times, requiring its special treatment. In fact, almost every invention and modern improvement has its concomitant disease, and almost every year brings some new disease of a serious nature with which the modern physician must cope. And he can't cope unless by constant study he prepares himself to do so. No physician should offer his services to the public unless he is willing to spend a reasonable part of his time in keeping abreast with his profession.

